# Potential and Future Therapeutic Applications of Eicosapentaenoic/Docosahexaenoic Acid and Probiotics in Chronic Low-Grade Inflammation

**DOI:** 10.3390/biomedicines13102428

**Published:** 2025-10-04

**Authors:** Amedeo Amedei, Ingrid Lamminpää, Cinzia Parolini

**Affiliations:** 1 Department of Clinical and Experimental Medicine, University of Florence, 50139 Florence, Italy; ingrid.lamminpaa@unifi.it; 2 Department of Pharmacological and Biomolecular Sciences, Rodolfo Paoletti, Università degli Studi di Milano, 20133 Milano, Italy

**Keywords:** cardiovascular disease, docosahexaenoic acid, eicosapentaenoic acid, microbiota, probiotics, specialized pro-resolving mediators

## Abstract

Nowadays, two major pathways seem to be responsible for the development and progression of atherosclerosis, namely, high levels of low-density lipoprotein-cholesterol (LDL-C) and low-grade vascular inflammation. Indeed, the concentration of C-reactive protein (CRP), mirroring low-grade systemic inflammation, has been recognized as a more powerful determinant of recurrent cardiovascular (CV) events, death, and all-cause mortality than LDL-C levels. Gut microbiota (GM) dysbiosis is a causal factor for the development of different inflammatory-based pathologies, such as CV disease (CVD). In addition, pre/probiotics showed beneficial effects on GM dysbiosis, by influencing both inflammation and immunity. It has been well documented that eicosapentaenoic acid (EPA) and docosahexaenoic acid (DHA) exert triglyceride (TG)-lowering and antithrombotic effects and play a seminal role in the resolution of inflammatory processes. We showed the recent studies indicating the relationship between pharmacological reduction in inflammatory cytokines and CV outcomes. The principal aim of our review is to highlight the anti-inflammatory and immune-modulatory activities of GM, EPA, and DHA. Then, we pointed out how developing patient-specific pre/probiotic and EPA/DHA interventions alongside the standard of care (SOC) is needed in order to answer several of the questions raised, ranging from diminishing drug toxicity to including frailty individuals. Therefore, hypothetical tailored clinical studies are presented, aiming to treat all the patients at high-risk of CV events, as well as aged people.

## 1. Introduction

Nowadays, two major pathways seem to be responsible for the development and progression of atherosclerosis, namely, high levels of low-density lipoprotein-cholesterol (LDL-C) and low-grade vascular inflammation [1,2]. In this scenario, LDL-C decrease is a seminal goal in preventive cardiology [3]. Meanwhile, in patients in secondary prevention who are taking a high dose of statins, the concentration of C-reactive protein (CRP), mirroring low-grade systemic inflammation, is a more powerful determinant of recurrent cardiovascular (CV) events, death, and all-cause mortality than LDL-C levels [4]. These data come from a recent collaborative analysis of three randomized clinical trials, PROMINENT, REDUCE-IT, and STRENGTH [4]. In these studies, patients with, or at high risk of, atherosclerotic CV disease (CVD), were treated with statins [5] and penafibrate (PROMINENT, *n* = 9988) [6] or AMR101 (icosapent ethyl) (REDUCE-IT, *n* = 8179) [7] or Epanova [a combination of eicosapentaenoic acid (EPA) and docosahexaenoic acid (DHA)] (STRENGTH, *n* = 13,078) [8]. The residual inflammatory risk (measured by CRP) was significantly associated with major CV events and CV and all-cause mortalities. Conversely, the relationship of residual cholesterol risk (measured by LDL-C) was neutral for major CV events and did not achieve statistical significance for CV and all-cause deaths [4]. These correlations were recently confirmed in statin-treated patients with well-controlled LDL-C and atherosclerotic CVD [9]. Therefore, in the setting of secondary prevention, clinicians must measure both CRP and LDL-C concentrations to better understand the true residual biological risk of their patients, aiming at the best “personalized medicine”. Furthermore, these findings underscore the need for therapeutic strategies targeting residual inflammatory risk alongside LDL-C lowering.

It is well documented that gut microbiota (GM) dysbiosis, namely, alterations in the composition and function of the intestinal microorganisms, is associated with different inflammatory-based pathologies [10,11]. Many studies have shown beneficial effects of pre/probiotics on GM dysbiosis, by modulating both inflammation and immunity [12,13]. Moreover, several decades of research have documented that EPA and DHA exert triglyceride (TG)-lowering and antithrombotic effects [14,15] and play a relevant role in the resolution of inflammatory processes, being the precursors of specialized pro-resolving mediators (SPMs) [16].

Since the residual inflammatory risk is pervasive and underappreciated [17], even among patients following guideline-directed treatments, we have reported the recent studies indicating that “lower is better” not only for LDL-C, but also for inflammation. This review aims to revise the role played by GM, EPA, DHA, and SPMs on immunity and inflammation. Furthermore, we have focused our revision on studies evaluating the efficacy of the combination between EPA/DHA and pre/probiotics. Finally, we identified limitations and raised new questions.

Therefore, further tailored trials are needed for overcoming limitations and answering the questions raised, ranging from diminishing the drug toxicity to including frailty subjects. Keeping in mind these goals, we depicted hypothetical clinical studies as “proof-of-concept” that pre/probiotics plus EPA/DHA/SPMs can positively impact the inflammatory status and, hopefully, the CVD outcomes of the patients.

## 2. Methodology

We carried out a narrative review of the English language literature. The usage of MeSH tool in PubMed allowed us to browse through NLM databases. Through this tool, we were able to refine our search and emphasize the relevant studies. The following search terms were combined: “eicosapentaenoic acid” OR “EPA” OR “docosahexaenoic acid” OR “DHA”AND “mice” OR “mouse” OR “rat” OR “rodent” OR “clinical studies” OR “human studies” AND “LDL” OR “triglycerides” OR “HDL” OR “atherosclerosis” OR “cardiovascular disease” OR “calcific aortic valve disease” OR “diabetes” OR “inflammation” AND “gut microbiota” OR “microbiota composition” OR “gut microbiota diversity” OR “gut microbiota dysbiosis” AND “probiotics” OR “prebiotics” OR “synbiotics”. The search was updated until July 2025.

## 3. Inflammation and Cardiovascular Disease

The accumulation of pro-inflammatory lipoproteins (mainly LDL) in the vessel wall predisposes the release of free cholesterol that can self-aggregate into crystalline forms. This eventually triggers the activation/dysfunction of endothelial cells as well as the inflammatory process, leading to the early steps and progression of atherosclerosis [18,19,20,21]. Different studies have documented that the endothelial dysfunction is followed by the activation of NLRP3 [nucleotide-binding oligomerization domain (NOD)-like receptor (NLR) family pyrin domain containing 3] inflammasome and the production of inflammasome-dependent cytokines, including interleukin (IL)-1beta and IL-18 [22,23,24]. IL-1beta locally promotes coagulation factors and platelet activation, thus inducing plaque rupture and thrombosis. At system levels, it stimulates IL-6 synthesis with hepatic production of CRP, which maintains a vicious cycle responsible for increasing CV events [2,25]. Conversely, IL-18 cooperates with IL-12 in stimulating the production of interferon (IFN)-gamma [26] from T and natural killer (NK) cells, causing NK cell activation, T helper (Th) 1 cell skewing, and upregulation of antigen presentation [27]. Several diseases are driven by elevated IL-18 levels, including multiple sclerosis, inflammatory bowel disease, and rheumatoid arthritis [17,28,29]. However, different studies suggest seminal physiological functions for IL-18. Therefore, IL-18 possesses both harmful and protective roles in inflammatory pathologies [30].

Calcific aortic valve disease (CAVD) is the most common valve pathology worldwide, and in developed countries, following coronary heart disease and hypertension, is the third most prevalent CVD [31]. CAVD is the result of the transition from aortic valve sclerosis, characterized by thickening of the aortic valve and mild calcification, to aortic valve stenosis, where the aortic valve leaflet thickening and calcification progress, eventually leading to obstruction at the valve level [32,33]. Therefore, patients may experience life-threatening outcomes, such as left ventricular hypertrophy, heart failure, and premature mortality [34]. Epidemiological surveys have shown that different degrees of aortic valve sclerosis are present in 25% of individuals aged 65 years, with a prevalence rate of 54.4% among those aged 85 years old or older [35]. Therefore, CAVD is emerging as an urgent global public health issue [36,37]. Like atherosclerosis, age, gender (male), body mass index, smoking, hypertension, dyslipidemia, diabetes, infections, and kidney disease are risk factors for this illness [38,39,40]. In addition, CAVD causes dramatic hemodynamic disturbance, i.e., shear stress, which may influence valve inflammation, immune cell function and disease progression [41], according to an observation of CRP elevated levels in CAVD patients [42]. In line with this data, a recent experimental study demonstrated a novel mechano-sensing mechanism of shear-inducing CRP dissociation, linking CAVD and inflammation [43]. Of note, statins do not effectively halt CAVD progression [44], and no established medical treatments have been able to delay the advancement of this pathology in asymptomatic patients up to now [41].

The publication of randomized and placebo-controlled trials showing that negative modulation of the NLRP3 inflammasome/IL-1beta/IL-6 axis lowers CV events rates among patients already taking guideline-directed medical care gives us new therapeutic options. In the CANTOS study, the IL-1beta inhibitor canakinumab significantly decreased major adverse CV event rates by 15 and 17%, without a clear modification of LDL-C and apolipoprotein (apo)B levels or blood pressure [45]. Of note, the Cardiovascular Inflammation Reduction Trial (CIRT) demonstrated no benefit in terms of CV event reduction with a non-specific anti-inflammatory drug, namely, methotrexate, that had no effects either on circulating concentrations of IL-1beta, IL-6, or CRP [46]. However, oral low-dose colchicine (0.5 mg/d), a microtubule polymerization inhibitor, had proven efficacy in lowering the rates of major CV events by 31% among patients with stable atherosclerosis (LoDoCo2) [47] and by 23% among those following recent myocardial infarction (COLCOT) [48].

**Figure 1:**
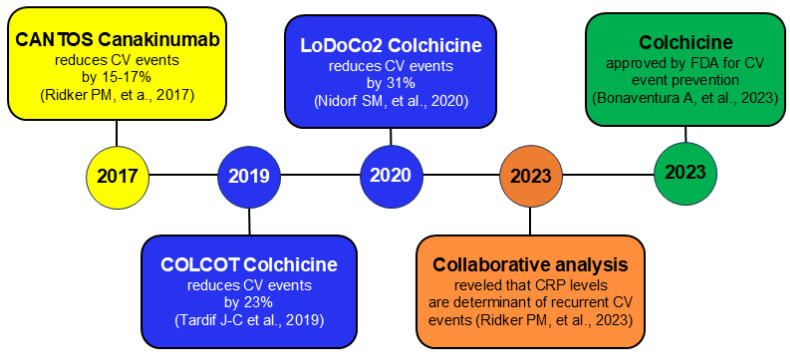
The flow chart represents clinical studies with Canakinumab [45] and Colchicine [47,48], the collaborative analysis [4] and the FDA approval of Colchicine [49]. CV, cardiovascular; FDA, U.S. Food and Drug Administration.

Therefore, the U.S. Food and Drug Administration (FDA) in 2023 approved low-dose colchicine as the first anti-inflammatory therapy to be used in combination with statins “to reduce the risk of myocardial infarction, stroke, coronary revascularization and CV death in adult patients with established atherosclerotic disease or with multiple risk factors for CVD” (Figure 1) [49].

In all the clinical studies, the colchicine was well tolerated, and serious adverse effects were similar with those reported in the placebo groups. However, based on the pharmacokinetic and pharmacodynamic profile of the colchicine, this drug is contraindicated in patients with severe kidney or hepatic dysfunction and should be temporarily discontinued when taking medications that inhibit the cytochrome 450 mixed-function oxidase (CYP)3A4 and P-glycoprotein metabolism pathways [45,47,48].

Before the above-mentioned clinical trials, the only intervention that could be suggested to the patients with elevated CRP was a more dramatic lifestyle change. Therefore, these data strengthened the need for looking for nutraceuticals [13,14,16], to lower the residual inflammatory risk, aiming at reducing the side effects and limitations of the standard of care (SOC). These nutraceuticals eventually might lead to the possibility of treating all the patients at high risk of CV events, as well as aged people, characterized by a chronic low-grade inflammatory status. Consequently, new, well-designed trials should be planned and performed considering (i) the duration of the study; (ii) the overall clinical parameters of the subjects recruited (e.g., lipid and microbiota profiles, kidney and liver functionality); and (iii) the composition of the control comparator.

## 4. GM–Inflammation–Immunity Axis

As previously reported, the gastrointestinal tract hosts the largest population of microorganisms in the body, namely GM. The microorganisms in this intestinal microbial community interact with one another and with epithelial and mucosal immune cells, aiming at maintaining immunological homeostasis. This interplay ensures prevention of the loss of immune tolerance, and maintenance of healthy levels of inflammation [11]. In addition, GM acts as an endocrine organ, producing molecules able to interact with the host physiology and triggering responses at local and distant levels. Alterations of this equilibrium, whether due to genetic or environmental insults, result in critical and far-reaching alterations in intestinal and extra-intestinal organs, such as the liver, lung, heart, and the central nervous system [50]. Indeed, GM dysbiosis has been associated with chronic low-grade inflammation and eventually with the etiology of chronic non-communicable diseases [11]. Divergent metabolites drive the interplay between the host and its microbiome. Specifically, short-chain fatty acids (SCFAs), produced by bacteria from the fermentation of fibres, trimethylamine (TMA) derived from the bacteria fermentation of L-carnitine and choline, bile acids produced in the liver and transformed by GM, and the tryptophan metabolites [13].

The major SCFAs encompass acetate (C2), propionate (C3), and butyrate (C4), which account for about 80% of all SCFAs. In the colon, acetate, propionate, and butyrate are present in a molar ratio of about 3:1:1, respectively [51]. Of note, the gut is the main producer of SCFAs, and so their concentration in the portal vein is five times greater than that found in peripheral venous blood [51]. Metagenomic analyses [52] and the gutSMASH algorithm [53] have identified that the synthesis of single SCFAs can be assigned to specific microbial phyla in the human GM. For example, acetate can be produced through two diverse pathways: (1) from pyruvate by enteric bacteria, including *Ruminococcus* spp., *Prevotella* spp., *Akkermansia muciniphila*, *Blautia hydrogenotrophica*, *Clostridium* spp., and *Streptococcus* spp. [54,55] and (2) from acetyl-CoA by acetogenic bacteria using the Wood-Ljungdahl pathway [56]. In line with these data, Nogal et al. demonstrated that the impact of GM composition and diversity on circulating acetate concentrations in a large population-based cohort, with different gut bacterial genera, was associated with either higher or lower acetate levels. In addition, serum acetate concentrations were inversely correlated with the amount of visceral fat, eventually positively influencing different cardiovascular disease risk factors [57].

SCFAs influence several crucial functions for the eukaryotic host, ranging from being an energy source for intestinal epithelial cells to exerting anti-inflammatory properties on immune cells (neutrophils, macrophages, and effector T cells) and promoting the peripheral production of regulatory T cells (Tregs) [13,58]. Their ability to function as histone deacetylase (HDAC) inhibitors is partially responsible for this effect, resulting in epigenetic changes that promote the growth and function of Tregs [58,59]. Indeed, butyrate can stimulate acetylation of the Foxp3 gene, which is a key transcription factor for Tregs [58,59]. Meanwhile, a recent study discovered a new immune-mediated pathway linking propionic acid with intestinal expression of the cholesterol transporter Niemann-Pick C1-like 1 (Npc1l1) and cholesterol homeostasis [60]. In detail, Haghikia et al. found that treatment with propionic acid lowers serum total and LDL-C concentration in both mice and patients. Moreover, in the mouse model used (namely, apoEKO), propionic acid reduced intestinal cholesterol absorption and atherosclerotic lesion burden. These effects were, at least in part, a consequence of the propionic acid’s ability to increase the number of Tregs and the IL-10 levels in the intestinal milieu, eventually leading to a downregulation of Npc1l1 gene expression [60].

Moreover, the consumption of diets rich in meat (such as the Western-type diet), which is a source of L-carnitine and choline, are directly correlated with the levels of TMA-N-oxide (TMAO), the product of the oxidation of TMA by the enzymatic activity of the liver flavin monooxygenase 3 (FMO3) [61]. Experimental and human studies have demonstrated that TMAO is atherogenic [61,62]. Moreover, Zhu et al. showed that microbial transplantation of a high TMA-producing bacteria could transmit TMAO production and promotion of thrombosis into recipient germ-free mice [63]. Elevated TMAO levels have been shown to predict a future risk of major adverse cardiac events, an increased CVD prevalence, and have been associated with the number of diseased coronary vessels [61,64,65]. In addition, the TMAO pathway has been linked to cardiac hypertrophy and fibrosis, chronic kidney disease, type2 diabetes, and obesity [66,67,68,69,70,71,72]. However, the molecular mechanisms behind these effects are not fully elucidated yet, mainly due to the lack of a recognized receptor or chemical sensor for TMAO. In vitro and in vivo studies have demonstrated that TMAO may (1) increase thrombin-induced Ca^2+^ release, enhancing platelet hyperreactivity [73]; (2) promote vascular inflammation by inducing the activation of mitogen-activated protein kinases (MAPKs) and nuclear factor kappa B (NF-kB) [74] as well as the NLRP3 pathways [75,76], in endothelial and smooth muscle cells; (3) activate the TGF-beta/SMAD3 (transforming growth factor/Small mother against decapentaplegic) signalling pathway, starting profibrotic processes in the heart and kidney [68]; (4) inhibit the reverse cholesterol transport, at least in part, by downregulating the hepatic expression of cholesterol 7alpha-hydroxylase (CYP7A1), the rate-limiting enzyme in bile acid synthesis, and some bile acids transporters [62]; and (5) stimulate macrophage cholesterol accumulation by enhancing cell surface expression of fatty acid transporter/scavenger receptor class B member 2 (Cd36/Sr-b2) and scavenger receptor A (Sra) [62].

Primary bile acids, released into the duodenum from the gall bladder, facilitate the absorption of dietary lipids and lipophilic vitamins. In the colon they are metabolized to secondary bile acids by GM, which encodes the enzymes involved in this unique microbial modification [11]. Of note, primary and secondary bile acids act as signalling molecules by interacting with host bile receptors, farnesoid-X-receptor (FXR), vitamin D3 receptor (VDR) and G-protein-coupled bile acid receptor (TGR5), localized on monocytes, macrophages, and dendritic and Kupffer cells. These activated receptors modulate the expression of pro-inflammatory genes through the NF-kB signalling pathway [13].

Tryptophan is an essential aromatic amino acid, recognized as a biosynthetic precursor of many microbial and host metabolites [77]. Specifically, in the gastrointestinal tract, dietary tryptophan can be directly transformed into indole and its derivates, which are ligands of the aryl hydrocarbon receptor (AhR) [78]. AhR signalling is considered a critical component of the immunity at barrier sites and, therefore, of the intestinal homeostasis, by modulating epithelial renewal, barrier integrity, and many immune cell types [79]. In addition, GM influences the kynurenine-producing IDO (indoleamine 2,3-dioxygenase) pathway, which plays a critical role in inflammatory mechanisms, immune responses, and neurobiological functions [80]. Finally, enterochromaffin cells produce a significant amount of the total body of serotonin (5-HT), including the plasma 5-HT [81]. It has been demonstrated that the 5-HT synthesis is under GM control, specifically mediated by SCFAs [82,83]. Gut-produced 5-HT is a pivotal gastrointestinal signalling molecule because it regulates intestinal peristalsis and motility, platelet aggregation, nociceptive neuron activation, immune responses, bone development, and cardiac functions [83]. 5-HT exerts these functions interacting with different receptors, classified into seven families and including at least 15 subgroups [84], expressed on enterocytes [85], enteric neurons [86], and immune cells [87].

In this scenario, diet is a critical environmental factor which impacts GM composition and activity and, vice versa, the nutrition value of food is, at least in part, influenced by the GM composition [11]. The Western lifestyle, including over-nutrition and sedentary behaviour, can lead to excess fat deposition and cause lipid-engorged hypertrophic white adipocyte’s expansion [88,89]. Altogether, these events stimulate the secretion of pro-inflammatory cytokines, such as tumour necrosis factor (TNF), IL-1beta, and IL-6, and mobilization of free fatty acids (FFAs), from adipose tissue into circulation [90]. These high levels of circulating FFAs are responsible for the ectopic accumulation of lipids in the bone marrow and thymus, the primary organ of the immune system [91]. In fact, this chronic exposure to an abnormal metabolic milieu leads to a dramatic imbalance of innate and adaptive immunity, eventually producing a state of chronic low-grade inflammation. In adipose tissue and in peripheral blood leukocytes from obese subjects, higher levels of pro-inflammatory lipid mediators compared with those of SPMs were found [92]. Notably, it has been suggested that this pro-inflammatory state observed in obese individuals is driven by the GM dysbiosis [93], due to the consumption of diets rich in fat and sugar and low in fibres. In fact, these diets are associated with low SCFAs and Bifidobacterium amounts, and a decrease in the Roseburia/Eubacterium rectale group [94]. On the other hand, prebiotics can selectively promote the growth of beneficial genera (Bifidobacterium and Roseburia), leading to increased production of SCFAs [95].

Based on these data, GM metabolites, mainly SCFAs and TMAO, could represent a target to positively impact the development/progression of CVD. However, many results come from experimental studies that have not been completely confirmed by human trials. Therefore, future and tailored experiments are needed to resolve these conflicting results, eventually aiming to unveil the causal relationship between GM and host metabolism.

## 5. EPA, DHA and SPMs

Recent reviews have described the pleiotropic effects of EPA and DHA, and those of the SPMs, endogenously synthesized from EPA, docosapentaenoic (DPA), and DHA [16,96,97]. Briefly, besides the well-documented TG-lowering effect [14,98], EPA and DHA exert anti-inflammatory activities by inhibiting the cell-surface expression of adhesion molecules and the production of inflammatory cytokines (i.e., TNF-alpha, IL1-beta, and IL-6) and COX-2 metabolites [99,100]. Indeed, EPA and DHA can dampen inflammatory signalling via the NF-kB pathway [101]. Three diverse mechanisms have been suggested: (1) activation of peroxisome proliferator-activated receptor (PPAR)-gamma, which physically interacts with the dimeric form of NF-kB, preventing its nuclear translocation; (2) interference with lipid raft formation in the membrane of inflammatory cells; and (3) binding to G-protein-coupled receptor (GPCR)120, also known as FFA receptor 4 (FFAR4) [98].

As reviewed by Bhat et al. [102], earlier studies on CV outcomes (namely, GISSI-Prevenzione [103] and JELIS [104]) demonstrated the beneficial effects of EPA and DHA. However, subsequent clinical large-scale trials performed to evaluate diverse doses and formulation of EPA and DHA, alone or in combination, did not confirm the previous results. These included OMEGA [105], SU.FOL.OM3 [106], ORIGIN [107], Risk and Prevention study [108], ASCEND [109], VITAL [110], STRENGTH [8], and OMEMI [111], who found no significant difference in the primary outcome compared to the placebo. However, in all studies, the amount of EPA and DHA was lower than that used in the positive trials. Consequently, the REDUCE-IT trial was designed with the administration of 4 g of purified EPA (icosapent ethyl) [7]. The primary outcome (a composite of CV death, non-fatal myocardial infarction, non-fatal stroke, coronary revascularization, or unstable angina) was significantly decreased in the EPA group compared to the placebo (17.2% vs. 22%, *p* < 0.001) [7].

Therefore, after the first FDA approval for the prescription of EPA/DHA (up to 3 g/d) in severe hypertriglyceridemic patients (>500 mg/dL) [112], in 2022, icosapent ethyl was approved by the FDA for CV risk reduction in high-risk patients [113]. Furthermore, a recently published randomized clinical trial (namely, RESPECT-EPA), was designed to assess whether icosapent ethyl could impact the recurrence of CV events in statin-treated patients who have a low baseline EPA/AA (arachidonic acid) ratio [114]. Icosapent ethyl treatment decreased the cumulative primary end point, even though this effect did not reach statistical significance. However, this treatment was significantly (*p* = 0.031) associated with a diminished risk of the secondary end point, such as sudden cardiac death, myocardial infarction, unstable angina, or coronary revascularization. In addition, the subgroup of patients who responded to icosapent ethyl treatment or had an increase in the EPA/AA ratio, showed fewer events compared with the patients enrolled in the placebo group (*p* = 0.021 and *p* = 0.009 for primary and secondary endpoints, respectively) [114].

A systematic review and meta-analysis published in 2021 investigated the effectiveness and safety of EPA and DHA on fatal and non-fatal CV events [115]. The data documented the moderate efficacy of EPA and DHA to reduce CV mortality and outcomes. The existence of relevant differences on the effects exerted by EPA and DHA on membrane structure, inflammatory cascade, lipid oxidation, endothelial function, and tissue distribution, associated with their divergent chemical structure, was also highlighted [115]. Of note, once in the body, EPA can be converted into DHA. The rate-regulating enzyme of this conversion is the delta-6 desaturase, encoded by the FADS2 gene [116]. Numerous single nucleotide polymorphisms (SNPs) have been identified in the human *FADS*-gene cluster [117]. Overall, the more common alleles were associated with higher serum concentrations of the products of EPA desaturation and vice versa [118,119].

SPMs, produced by the action of cyclooxygenase (COX), lipoxygenase (LOX), and CYP450 enzymes, are a family of molecules that includes resolvins (Rvs), protectins (such as, neuroprotectins, PD1/NPD1), maresins (MaRs), and the novel cysteinyl-SPMs (cys-SPMs) [120]. SPMs play a critical role in the “resolution phase” of the inflammatory response [120]. In detail, these mediators may (1) limit granulocyte chemotaxis and infiltration; (2) stimulate the M2 macrophage polarization, phagocytosis, and efferocytosis; (3) accelerate wound healing; (4) reduce the production of pro-inflammatory cytokines (TNF-alpha and IL-1beta) and lipid mediators (prostaglandins and leukotrienes); (5) promote the Treg response and release of IL-10; and (6) diminish platelet aggregation and inflammasome formation [120,121,122].

Of note, in the presence of aspirin and statins (as occurred in the clinical studies of secondary CVD prevention [97]), an increased synthesis of some SPMs, such as lipoxins, Rvs, and protectins, have been detected via acetylation or S-nitrosylation of the enzyme COX-2, respectively [123]. Therefore, this could represent an additional mechanism to justify the anti-inflammatory and pro-resolving effects observed with aspirin and statins treatments [124,125,126,127].

These data, together with an emerging body of experimental evidence, strengthened the hypothesis that SPMs may exert protective roles in atherosclerosis and related CVD [128]. Administration of RvE1 to apoE*3 Leiden mice fed a high-fat/high-cholesterol diet was associated with a significant reduction in atherosclerotic plaque development, together with the decreased gene expression of pro-inflammatory cytokines (e.g., TNF and IFN-gamma) [129]. D-series resolvins (namely, RvD1, RvD5n-3 DPA, and RvD2) and MaR1 had proven efficacy in promoting plaque stability as well as in decreasing atherosclerotic lesion size and leukocyte/platelet activation in high-fat fed LDLR-null [130,131] and apoEKO mice [132], respectively.

It has been demonstrated that SPMs act through distinct GPCRs, ALX/DRV1/GPR32 [133], DRV2/GPR18 [134], GPR37 [135], LGR6 [136], ERV1/ChemR23, and BLT1 [137]. In hyperlipidemic mice [138] the lack of the receptor ERV1/ChemR23 was associated with proatherogenic signalling in macrophages, increased oxidized-LDL uptake, reduced phagocytosis, and larger atherosclerotic plaque volume and necrotic core formation compared with those observed in wild-type animals [139]. Moreover, ERV1/ChemR23 was detected in human atherosclerotic specimens, and its mRNA expression levels were found to be higher in lesions derived from statin-users compared with non-users [139]. Since DRV2/GPR18 play a seminal role in the inflammatory “resolution phase” [140], the RvD2-GPR18 axis may represent a new target aiming at developing “pro-resolution” therapies for inflammatory-based pathologies [141,142].

However, the translation of these experimental results into improving human health has been challenging. Intravenous or intraperitoneal administration of SPMs is not feasible for humans, as it is for rodents. Oral SPMs delivery is not reasonable due to their relatively short half-life in biological fluids [143]. Several strategies have been investigated to overcome this relevant issue [144]. For instance, the SPMs pharmacokinetics may be improved by using a metabolically more stable form of SPMs or delivery system that could mask the active molecules before reaching the tissue target [145,146]. In addition, data from patients affected by inflammatory-correlated pathologies, such as diabetes, metabolic syndrome, and inflammatory bowel disease, suggested that the endogenous SPM-producing machinery may be dysfunctional under certain human illnesses [147]. Elajami et al. documented that specific SPMs, present in measurable levels in healthy controls, were undetectable in coronary artery disease patients, and treatment with Lovaza (4 capsules daily containing 3360 mg EPA and DHA) for 1 year seemed to restore the formation of these SPMs [148].

Altogether, these data suggest that SPMs could represent new therapeutic strategies to promote the resolution of the inflammatory process, thus reducing the residual inflammatory risk. However, these results come from experimental/preliminary evidence and will require other pre-clinical and clinical studies to achieve a final statement.

## 6. Evidence of the Interplay Among EPA, DHA, Prebiotics/Probiotics and GM

Both experimental and clinical studies have shown the ability of EPA/DHA to affect GM by (1) impacting the diversity and abundance of its community (specifically, decreasing the Firmicutes/Bacteroidetes ratio and the levels of Coprococcus and Faecalibacterium, and increasing the amount of butyrate-producing bacterial genera, i.e., Bifidobacterium, Lachnospira, Roseburia, and Lactobacillus) [149,150]; (2) modulating levels of pro-inflammatory molecules, such as IL-17 and lipopolysaccharides (LPS) [95,97,151]; and (3) altering the concentrations of SCFAs and their salts [95]. For instance, an increase in cecal levels of acetate and butyrate and the Clostridiaceae were detected in mice fed a diet enriched with EPA/DHA compared to mice fed a diet deficient in EPA/DHA [152]. In addition, this increased abundance of SCFA-producing bacteria was confirmed in two randomized clinical trials with patients treated with EPA/DHA, and the *Coprocossus* amount was inversely associated with the concentration of pro-atherogenic lipoprotein (such as very-low density lipoproteins and LDL) [150,153]. Coherently, EPA/DHA displays beneficial CV activity through restoring the microbiota composition and stimulating the production of anti-inflammatory metabolites, such as SCFAs, in a high-risk population [145]. In line with these data, a fecal metagenome characterization of healthy, pre-diabetic, and type2 diabetic females highlighted that one gene cluster from *Clostridiales* was inversely correlated with TG and directly associated with high-density lipoprotein-cholesterol (HDL-C) levels [154]. Moreover, in subjects with a high-risk CVD lipid profile (namely, high TG and low HDL-C concentrations) a low microbial diversity was observed, whereas no correlations were found between GM variation and total or LDL-C concentrations [155]. Altogether, these results demonstrated that EPA/DHA can act as prebiotics, exerting a synergic effect on the microbiota community, and modulators of CVD risk factors.

Diverse studies have demonstrated that the consumption of prebiotics, probiotics, and synbiotics (a combination of prebiotics and probiotics) may have a beneficial impact on human health [156,157]. In fact, the term “probiotic” refers to live microorganisms that confer a health benefit on the host who consumes them in adequate amounts [158]. For example, the probiotic bacteria Lactobacilli and Bifidobacteria have been associated with improved intestinal barrier function and integrity through diverse mechanisms, such as Toll-like receptor2 (TLR2)-mediated immune modulating and anti-inflammatory effects, promoting the production of butyrate and the expression of tight junction proteins, such as ZO-1 and occludin [159]. Conversely, the definition of prebiotic is “non-digestible food ingredients that beneficially influence the host by selectively stimulating the growth and/or the activity of one or a limited number of bacteria in the colon, thus improving host health” [160].

**Figure 2:**
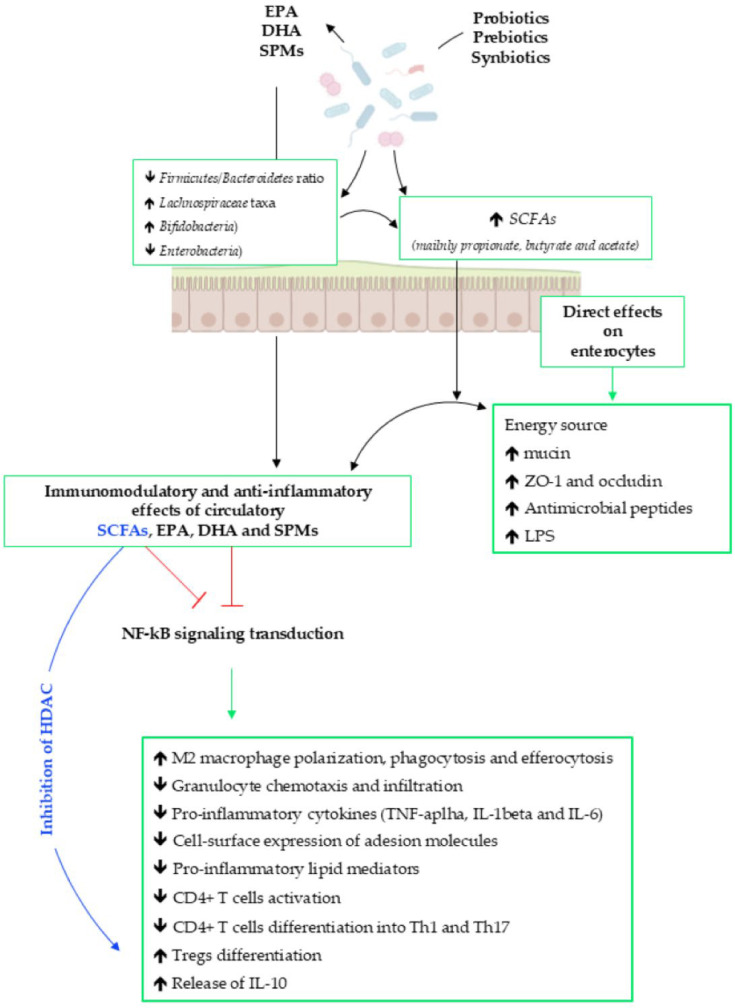
The local and peripheral immunomodulatory and anti-inflammatory effects of DHA, EPA, SPMs, prebiotics, probiotics, synbiotics, and GM metabolites. Please refer to the text for the meaning of all abbreviations used in the figure. [Created in BioRender.com].

Finally, it is important to remember that the specific beneficial taxa (mainly, *Bifidobacterium*, *Lactobacillus*, and *Akkermansia*) may strongly impact the bioavailability and function of EPA/DHA, by influencing their absorption and metabolism through the upregulation of key enzymes, such as, desaturase and elongase [161]. On the other hand, prebiotics can selectively promote the growth of beneficial genera (*Bifidobacterium* and *Roseburia*), leading to the increased production of SCFAs and enhanced bioavailability of EPA/DHA [149,150]. These data highlight the complex interplay among GM, pre/probiotics, and EPA/DHA, eventually influencing host health, as shown in Figure 2.

Experimental studies performed in rodents demonstrated that the consumption of Bifidobacterium breve CECT7263, *Lactobacillus fermentum* CECT5716 [162], the probiotic formulation composed of *L. fermentum* 139, 263 and 296 [163], and the combination between prebiotic inulin and probiotic Lactobacillus casei was able to inhibit the CVD development (Table 1) [164].

This effect was associated with a reduction in the Firmicutes/Bacteroidetes ratio, an increase in the levels of Lactobacillus and *Akkermansia muciniphila*, the latter being beneficial bacteria for GM balance, and a decrease in plasma TMAO amounts. Moreover, a significant increase in the butyrate levels was observed, suggesting that these supplements may be able to modulate oxidative stress and inflammatory response [164,165]. A recent experimental study performed on healthy mice showed that early-life dietary interventions with a prebiotic, composed of a mixture of short-chain galacto-oligosaccharide (scGOS) and long-chain fructo-oligosaccharides (lcFOS) and/or EPA/DHA affected the cecal content microbial profile [166]. In detail, the combination increased the relative abundance of Allobaculum, S24-7 Unclassified, and Akkermansia, while reducing the abundance of the genera Oscillospira and Ruminococcaceae [166].

**Table 1:** Impact of probiotics, prebiotics, synbiotics, and EPA/DHA on GM and CV risk factors in rodents.

Study Type	Intervention	Main Effects	Refs.
Experimental (rats)		Protection of intestinal barrier	
	Synbiotics	Prevention of dysbiosis Prevention of endothelial dysfunction Decrease in high blood pressure	[162]
Experimental (rats)	Probiotics	Decrease in inflammatory response Increase in beneficial bacteria	[163]
Experimental (rats)		Reduction in the *Firmicues/Bacteriodetes* ratio and TMAO levels	
	Synbiotics	Increase in butyrate concentration and amount of *Lactobacillus* and *Akkemansia muciniphila*	[164]
		Reduction in the oxidative stress	
Experimental (mice)	Prebiotics plus	Increase in Alloculum S24-7 and *Akkemansia muciniphila*	[166]
	EPA + DHA	Reduction in Oscillospira and Ruminococcaceae	

TMAO—Trimethylamine N-oxide; EPA—Eicosapentaenoic acid; DHA—Docosahexaenoic acid.

Speckmann et al., aiming at overcoming the issues linked to the administration of SPMs, developed a synbiotic approach based on the discovery that Bacillus megaterium strains are potent producers of SPMs [147]. A double-blind, randomized, placebo-controlled 4-week intervention study was then conducted in middle-aged, healthy, but at-risk subjects [167]. The enrolled people received (1) the synbiotic omega 3 (SynΩ3) capsules, containing 1 billion colony-forming units of Bacillus megaterium, EPA + DHA (83.3 mg + 41.7 mg) and 27.5 microg selenium; (2) fish oil capsules (180 mg EPA, 120 mg DHA and 2.7 mg alpha-tocopherol); and (3) cellulose powder capsules, as a placebo (Table 2). This study confirmed the ability of SynΩ3 to significantly increase the plasma concentrations of SPMs compared with the fish oil group [167]. These results strengthen the concept that an altered or limited metabolic capacity of the body to synthesize SPMs can be compensated by suitable microbes or synbiotics [148].

Preliminary evidence showed that probiotics may impact the concentration of serum FFAs. Indeed, the administration of Lactobacillus gasseri SBT2055 was able to decrease the FFAs levels in hypertriglyceridemic patients (Table 2) [168].

In addition, the combination of dietary counselling and Lactobacillus rhamnosus GG and Bifidobacterium lactis Bb12 demonstrated the possibility of improving the profile of both the FFAs and different cytokines in the breast milk of pregnant women [169]. A recent randomized controlled trial was conducted in pregnant women with overweight and obesity [170]. The study showed that EPA/DHA administered from early pregnancy onward significantly increased their serum concentration (Table 2). Of note, an inverse association between CRP (reflecting the low-grade inflammation status) and EPA/DHA levels was detected (all r > −0.300, *p* < 0.01) [170].

## 7. Future Perspectives

**Figure 3:**
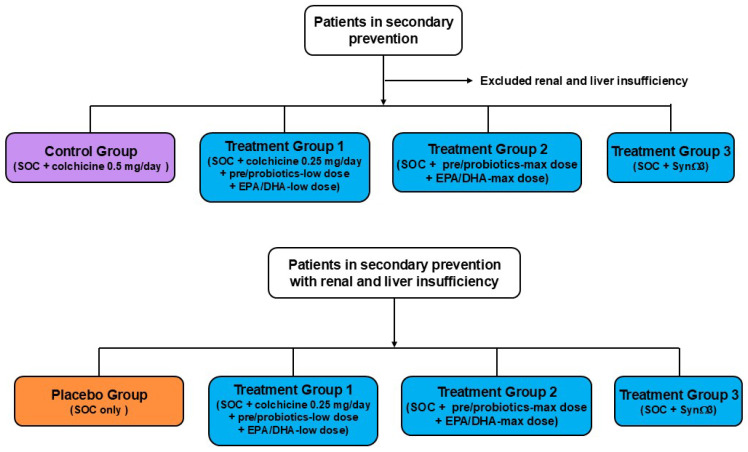
Patient flow of two hypothetical clinical trials to validate the anti-inflammatory effect of pre/probiotics and EPA/DHA. SOC, standard of care.

**Figure 4:**
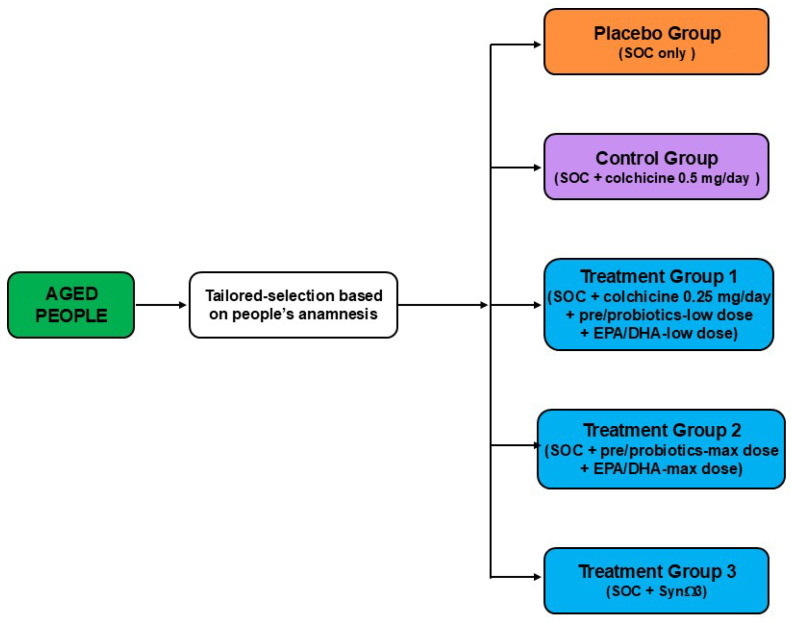
Patient flow of a hypothetical clinical trial to validate the anti-inflammatory effect of pre/probiotics and EPA/DHA. SOC, standard of care.

Based on these results, we can assess the existence of a dynamic interplay among gut microbial community, EPA/DHA, and modifiable CVD risk factors (such as LDL-C, HDL-C, and CRP). However, some questions are still without a clear answer, ultimately leading to the need for further studies. Indeed, some data are derived only from experimental studies and must be validated with human trials. To this aim, hypothetically, well-conducted and controlled clinical trials could be designed by adding pre/probiotics and EPA/DHA to the standardized therapeutic protocols, aiming at including all the patients at high risk of CV events (Figure 3), specifically those with functional alterations of kidneys and /or liver, as well as aged people (Figure 4).

Indeed, these future clinical studies could achieve a double aim. Firstly, further confirmation that lowering the inflammatory status results in a better outcome for patients in secondary prevention, overcoming the limitations due to the functional alterations of the kidney and/or liver (see, Treatments Group 1 in Figure 3) and genetic polymorphisms (FADS-gene cluster) (see, Treatments Group 3 in Figure 3). Meanwhile, these hypothetical trials that enrol aged people with chronic low-grade inflammation could decrease health costs by improving the quality of life of these frailty subjects.

## 8. Conclusions

Nowadays, high levels of LDL-C and low-grade vascular inflammation are the major determinant for the atherosclerosis development. In addition, the CRP concentration mirroring the low-grade systemic inflammation has been identified as a more powerful determinant of recurrent CV events, death, and all-cause mortality than LDL-C levels. Consequently, the FDA approved low-dose colchicine as the first anti-inflammatory drug to be used in combination with statins. However, colchicine is contraindicated in patients with severe kidney or hepatic dysfunction.

The hypothesis to improve the residual CVD risk with dietary interventions including EPA/DHA, and pre/probiotics has been preliminary validated in different experimental and clinical studies. However, further large, well-designed randomized trials are needed to clarify (1) the dose-dependent effects of EPA and DHA, separately and together; (2) the non-neutral mineral oil comparator [96]; and (3) the trend toward an increase in atrial fibrillation associated with EPA ethyl-ester monotherapy and mixed EPA/DHA formulations [171]. In addition, when discussing pre/probiotics, it is important to highlight that the efficacy of these substances can be conditioned by the strains and protocol used (such as quantity and length of the study), as well as by GM composition and the overall health condition of the subjects [12].

Finally, we highlighted how developing patient-specific pre/probiotic and EPA/DHA interventions alongside the SOC is needed to answer several of the questions raised, ranging from diminishing drug toxicity to including frailty subjects. In line with these premises, hypothetical tailored clinical studies have been presented, aiming to treat all the patients at high risk of CV events, as well as aged people. In detail, these hypothetical trials could help in unveiling the real efficacy of therapeutic tools targeting the resolution phase of the inflammatory process, eventually improving the residual inflammatory risk. Finally, these results will provide the clinician with different therapeutic options to treat all the different clinical situations, such as patients in secondary prevention with hepatic and/or liver dysfunction and frailty patients characterized by a state of chronic low-grade inflammation.

## Figures and Tables

**Table 2:** Impact of probiotics, prebiotics, synbiotics, and EPA/DHA on GM and CV health.

Study Type	Intervention	Main Effects	Refs.
Clinical trial	Probiotics plus		
	EPA + DHA	Increase in SPMs	[167]
Clinical trial	Probiotics	Reduction in FFA concentration	[168]
Clinical trial	Probiotics	Improvement of FFA and cytokine profile	[169]
Clinical trial	Probiotics plus	Reduction in CRP	[170]
	EPA + DHA	Increase in EPA and DHA levels	

EPA—Eicosapentaenoic acid; DHA—Docosahexaenoic acid; SPMs—specialized pro-resolving mediators; FFA—free fatty acid; CRP—C-reactive protein.

## Data Availability

No new data were created or analyzed in this study. Data sharing is not applicable to this article.

## Abbreviations

The following abbreviations are used in this manuscript:

Apo	Apolipoprotein
AA	Arachidonic acid
AhR	Aryl hydrocarbon receptor
CAVD	Calcific aortic valve disease
CV	Cardiovascular
CVD	CV disease
CRP	C-reactive protein
COX	Cyclooxygenase
CYP	Cytochrome 450 mixed-function oxidase
DHA	Docosahexaenoic acid
EPA	Eicosapentaenoic acid
FDA	U.S. Food and Drug Administration
FFAs	Free fatty acids
GPCR	G-protein-coupled receptor
HDL-C	High-density lipoprotein-cholesterol
IFN	Interferon
IL	Interleukin
LPS	Lipopolysaccharides
LOX	Lipoxygenase
lcFOS	Long-chain fructo-oligosaccharide
LDL-C	Low-density lipoprotein-cholesterol
MaRs	Marensins
NF-kB	Nuclear factor kappa B
NLRP3	Nucleotide-binding oligomerization domain (NOD)-like receptor (NLR) family pyrin domain containing 3
Tregs	Regulatory T cells
Rvs	Resolvins
5-HT	Serotonin
scGOS	Short-chain galacto-oligosaccharide
SCFAs	Short chain fatty acids
SPMs	Specialized pro-resolving mediators
TMA	Trimethylamine
TMAO	TMA N-oxide
TNF	Tumour necrosis factor
